# MM120 demonstrates no evidence of abuse potential in rodent preclinical studies

**DOI:** 10.1192/j.eurpsy.2025.2101

**Published:** 2025-08-26

**Authors:** J. Tripp, G. N. Smagin

**Affiliations:** 1 Mind Medicine, Inc., New York, United States

## Abstract

**Introduction:**

Generalized anxiety disorder (GAD) is a chronic and disabling disorder with an estimated lifetime prevalence of up to 7.8% in the US. MM120 (lysergide D-tartrate), a tartrate salt of D-lysergic acid diethylamide (LSD), is currently under development as a potential treatment for GAD. The mechanism of action (MOA) by which MM120 may elicit profound lasting psychological changes is not fully understood. Studies suggest that it has significant effects on functional brain activity across several networks, likely a result of serotonergic agonism mediated by 5HT2A activity and subsequent changes in neural network connectivity.

**Objectives:**

A series of Good Laboratory Practice (GLP) compliant studies were conducted to evaluate abuse potential of MM120.

**Methods:**

In vitro assessments of MM120, and its main metabolite 2-oxo-3-hydroxy LSD, receptor binding activity were performed using Eurofins CEREP BioPrint. To examine single dosing effects, MM120 was administered by oral gavage to male Sprague Dawley rats (n=6/group) at doses of 0 (vehicle only), 0.5, 2.0, and 6.0 mg/kg. A Modified Irwin test evaluated shorter acting drug effects at 15, 60, 120, and 240 minutes post-dosing compared to pre-dose baseline, with long-term, single dose neurological effects studied in a functional observation battery (FOB) on the day of dosing and 24 hours post dose. To compare potential chronic MM120 effects, male and female Sprague Dawley rats were dosed daily for four weeks via oral gavage at 0 (vehicle only), 0.5, 2.0, and 6.0 mg/kg (n=10-15/sex/group) followed by a 4-week recovery phase. FOB was performed pre-dose, dosing day 27, and day 25 of recovery phase. Additional assessments included toxicological and toxicokinetic evaluations.

**Results:**

Receptor binding assay confirmed MM120’s MOA is mediated by the serotonin system and hallucinogenic effects are driven by agonism at 5-HT2A and 5-HT2C receptors. MM120 demonstrated agonist activity at 5-HT1A, 5-HT1B, 5-HT1D, 5-HT5A, 5-HT6, and 5HT7 receptors and only nanomolar affinity at D2 dopaminergic receptors. Single dose MM120 at 0.5 mg/kg, had no effects on behavioral or physiological states. Following 6 mg/kg MM120, vocalization was recorded in 1/6 rats 2-4 h post dose, and incidences of mild piloerection were recorded in 3/6 rats from 4 to 24 h post dose. In the chronic study, there were no MM120-related changes in basic or fine movements, total ambulation, total rears, or total distance traveled.

**Conclusions:**

Binding studies suggest no indication for elements of abuse for MM120 even with binding at serotonergic and, to a smaller degree, dopaminergic receptors. in vivo studies with MM120 evaluated supratherapeutic doses and demonstrated no evidence of physical dependence or withdrawal after sustained, daily administration for four weeks.

**Disclosure of Interest:**

J. Tripp Employee of: Mind Medicine, Inc., G. Smagin Employee of: Mind Medicine, Inc.

